# The overlooked dimension of trauma: recognizing insidious trauma in intellectual disability

**DOI:** 10.3389/fpsyt.2026.1704086

**Published:** 2026-02-13

**Authors:** Ayelet Gur

**Affiliations:** Social Work Department, Tel Hai College, Kiryat Shemona, Israel

**Keywords:** insidious trauma, intellectual disability, marginalization, trauma, trauma informed care

## Introduction

This commentary argues for the recognition and application of insidious trauma theory to intellectual and developmental disability populations. While the trauma literature in intellectual disability has predominantly focused on discrete adverse events, this narrow conceptualization overlooks a pervasive form of trauma experienced through chronic devaluation, stigmatization, and systematic marginalization. This paper aims to illuminate evidence of ongoing psychological harm within existing research and advocate for expanded trauma frameworks that capture these overlooked experiences in intellectual disability populations.

## Conceptualizations of trauma

Traditional trauma conceptualizations focus on identifiable events meeting specific severity criteria. Levenson (1) defines trauma as exposure to extraordinary experiences posing physical or psychological threats and eliciting helplessness and fear. The DSM-5 exemplifies this event-focused approach, defining trauma as exposure to actual or threatened death, serious injury, or sexual violence through direct experience, witnessing, learning about events affecting close others, or repeated exposure to aversive details. PTSD diagnosis requires extreme events (excluding media exposure unless work-related) and specific symptom clusters: intrusive memories, avoidance, negative mood/cognitive changes, and altered reactivity (2).

The Substance Abuse and Mental Health Services Administration (3) bridges event-based and experience-based approaches, defining trauma as events or circumstances experienced as harmful or life-threatening with lasting adverse effects on functioning and well-being. SAMHSA’s framework emphasizes three elements, Event(s), Experience, and Effect, recognizing that traumatic impact depends on how individuals experience events, shaped by cultural beliefs, social supports, and developmental stage.

The concept of insidious trauma departs from traditional trauma paradigms, addressing the cumulative psychological impact of chronic oppression and marginalization experienced by non-dominant groups rather than focusing on singular catastrophic events. Developed by feminist psychotherapist Maria Root, insidious trauma is defined as an indirect trauma that shapes the subject’s worldview and sense of self, fundamentally associated with the devaluation of an individual’s identity. Laura Brown expanded this framework, defining insidious trauma as the traumatogenic effects of oppression that may not be overtly violent but harm the soul and spirit (4, 5).

Root (6) proposed that cumulative exposure to chronic devaluation and marginalization produces heightened vulnerability in marginalized individuals (as cited in Brown 7). This accumulated vulnerability can transform seemingly minor incidents into experiences that trigger full traumatic stress responses, including intrusive memories, avoidance behaviors, emotional numbing, and hyperarousal. Root (6) noted that the frequency of these experiences shatters an individual’s sense of safety and security and creates constant hypervigilance through activation of survival behaviors. Additional manifestations include internalized shame, development of defensive behaviors, inability to seek help due to cultural bias, emotional suppression, and deprivation of personhood (5). (8) advanced the concept of oppression artifact disorder, proposing that psychological distress patterns often diagnosed as personality pathology may more accurately reflect adaptive responses to chronic oppression embedded within institutional structures. This reconceptualization frames such responses as functional strategies for managing intense emotional distress while attempting to preserve necessary relationships with sources of that distress (as cited in Brown 7).

## Event-based trauma research in intellectual disability populations

The research on trauma in individuals with intellectual disabilities has primarily focused on event-based conceptualizations, examining discrete adverse experiences and their outcomes. This work has shown that individuals with intellectual disabilities are more vulnerable to traditional trauma exposures.

Studies consistently report higher rates of adverse childhood experiences (ACEs) among individuals with intellectual disabilities compared to the general population. Rose et al. (9) found that 72.1% of participants with disabilities experienced at least one ACE, compared to 60.8% of those without disabilities, with significantly higher odds of experiencing four or more ACEs. Berg et al. (10) reported that children with developmental disabilities were 28% more likely to report 1–2 adverse family experiences and 60% more likely to report 3+ adverse experiences than their non-disabled peers, highlighting a disproportionate burden of adverse events.

The literature links specific traumatic events to mental health outcomes. Research has shown connections between trauma exposure and conditions like psychosis, personality disorders, and increased aggressive behaviors. Notable predictive relationships include childhood sexual abuse and PTSD symptom severity, as well as ACEs and intimate partner violence in adulthood (11). Research has also examined behavioral and clinical manifestations of trauma. McNally et al. (11) noted that trauma responses in adults with intellectual disabilities often manifest behaviorally and emotionally rather than cognitively.

Environmental and contextual factors that heighten vulnerability to trauma have been studied extensively. Identified risk factors include a reduced ability to predict harm, inappropriate management of challenging behaviors, unnecessary restrictions, and environments inadequately adjusted to individual needs. Other factors include multiple stressful life events, lack of self-determination, dependency on others, and feelings of being different (11).

## Evidence of insidious trauma in the intellectual disability literature

Evidence of social identity damage, pathologization, and internalized devaluation is prevalent in research on how individuals with intellectual disabilities form their sense of self. Logeswaran et al. (12) found that most individuals recognize their disability label and experience significant negative emotions such as shame, embarrassment, anger, and frustration. These feelings are linked to perceptions of inability and negatively impact relationships and independence. Awareness of societal devaluation starts early, with young people in both mainstream and special schools recognizing societal stigma. This early awareness, along with negative emotional responses, suggests developmental identity damage consistent with insidious trauma.

Contributing to this identity damage, Dorozenko et al. (13) argued that the identities of people with intellectual disabilities are often pathologized, with their behaviors being interpreted as characteristic of their diagnosis rather than recognizing that these behaviors may be ordinary and normal. This pathologizing process systematically reduces individuals to their disability label, further compromising healthy identity formation. The psychological impact of such systematic identity reduction is evident when given voice in research, as people with intellectual disabilities directly describe the emotional consequences of these experiences. Roth et al. (14) found that individuals experience stigma with responses including avoidance, frustration, pain, and sadness.

Evidence also emerges through stigmatization by care providers and support systems. Research examining care provider attitudes reveals concerning patterns of stigmatization that may contribute to insidious trauma experiences. Pelleboer-Gunnink et al. (15) conducted a scoping review of 40 studies and found clear indications of stigmatization of people with intellectual disabilities by care providers, with stigmatization being more pronounced for those with high support needs. Anis and Nordin’s (16) systematic review of support staff attitudes documented skeptical views toward meaningful participation and independent decision-making among people with intellectual disabilities. Staff frequently assumed individuals were incapable of making independent decisions, reflecting systematic underestimation of capacity and agency consistent with the identity devaluation characteristic of insidious trauma.

Perhaps most significantly, the literature demonstrates the pervasive and chronic nature of devaluation experiences. The research consistently shows that devaluation experiences are pervasive patterns spanning multiple life domains rather than isolated incidents. Research reveals systematic skepticism about capacity across care relationships (16), awareness of societal stigma from childhood through adulthood (12), and structural segregation in employment, housing, and social systems (13). This pervasive and chronic nature aligns with insidious trauma’s emphasis on ongoing, systemic oppression rather than discrete traumatic events.

The patterns of devaluation documented in this literature can be understood through what Liasidou (17) describes as the relationship between ableism and trauma. Ableism—the stigmatizing attitudes, discriminatory structures, and devaluing social responses—represents the external mechanism through which insidious trauma occurs, while insidious trauma describes the internalized psychological harm experienced as a result. The systematic stigmatization, pathologization of identity, and chronic awareness of societal devaluation documented here represent manifestations of ableism that create the conditions for the ongoing identity devaluation characteristic of insidious trauma.

## Conclusion and implications

This examination reveals evidence of insidious trauma within intellectual disability populations, despite a lack of recognition in current research. Documented patterns of chronic devaluation, systematic stigmatization, pathologization of identity, and pervasive marginalization align with the concept of insidious trauma as ongoing oppression that shapes worldview and sense of self.

The lack of recognition of insidious trauma in intellectual disability populations represents a significant gap in research and practice. Recognition of this form of trauma is essential for developing comprehensive understanding of the psychological experiences of people with intellectual disabilities and moving beyond narrow event-based trauma conceptualizations that may inadequately capture their lived experiences.

Future research is urgently needed to systematically investigate insidious trauma in intellectual disability populations. However, such research must be accompanied by the development of methodologies that address the unique ethical considerations inherent in studying trauma experiences among vulnerable populations. This includes ensuring meaningful participation of people with intellectual disabilities as research partners rather than merely subjects, developing accessible research methods, and implementing safeguards that prevent further harm while gathering essential knowledge.

While comprehensive exploration of interventions is beyond this commentary’s scope, emerging trauma-informed care work offers relevant directions. These include systematic staff training as an ongoing process rather than one-time sessions (18), empowerment interventions promoting self-advocacy and choice (19), and structural policy review through a “trauma lens” (20, 21). However, current trauma-informed care frameworks have not been specifically developed to address insidious trauma. Substantial modification is needed to address the ongoing, systemic nature of insidious trauma, including innovative interventions that address both individual healing and systemic change rather than focusing solely on discrete traumatic events.

Recognition of insidious trauma in intellectual disability populations has the potential to transform both research and practice, moving toward more comprehensive understanding and more effective support for individuals who have experienced chronic marginalization and devaluation throughout their lives.
